# Poster Session II - A238 YOU ARE WHAT EATS YOU: EXPLORING MUCIN DEGRADATION BY THE IBD PATHOBIONT *CANDIDA ALBICANS*

**DOI:** 10.1093/jcag/gwaf042.238

**Published:** 2026-02-13

**Authors:** J A Sousa, I Yavuukhulan, G R Lunken, B Vallance

**Affiliations:** Gastroenterology, The University of British Columbia Faculty of Medicine, Vancouver, BC, Canada; Gastroenterology, The University of British Columbia Faculty of Medicine, Vancouver, BC, Canada; Gastroenterology, The University of British Columbia Faculty of Medicine, Vancouver, BC, Canada; Gastroenterology, The University of British Columbia Faculty of Medicine, Vancouver, BC, Canada

## Abstract

**Background:**

Bacterial dysbiosis is well known to contribute to the pathophysiology of Crohn’s disease (CD), however, less is known about the gut mycobiome. The CD fungal pathobiont *Candida albicans* has been shown to be increased in abundance and associated with disease severity in patients with CD. There is emerging evidence that *C. albicans* can degrade gut mucins, an important part of the intestinal barrier, but whether this activity is a significant contributor to disease pathology and how the gut environment contributes to this phenotype is unknown. Specifically, the increased oxygen levels seen in the intestines of patients with CD may enhance *C. albicans*’ mucin degradation and its growth as a facultative anaerobe. Understanding how *C. albicans* reacts to the intestinal environment of patients with CD, in terms of its mucin degradation capability, could reveal mechanisms of pathophysiology and ultimately lead to novel treatments.

**Aims:**

Determine the mucin degrading capability of *C. albicans* under both aerobic and anaerobic conditions.

**Methods:**

Growth curves were conducted using commercially available type II and III porcine gastric mucins (PGM) and bovine submaxillary mucins (BSM) as nutrient sources. Mucin degradation was assessed using an agarose plate-based assay and gel electrophoresis with subsequent immunoblotting. Invasion into the agarose-mucin matrix was determined through cross-sectioning of the agarose and depth of invasion quantification using ImageJ. Intracellular uptake of mucins was visualized using fluorophore conjugated PGM and confocal microscopy. Assays were conducted under both aerobic and anaerobic conditions.

**Results:**

*C. albicans* was able to grow on and degrade type II and III PGM but not BSM in the presence of an external carbon source (dextrose) suggesting mucins can only be utilized as a nitrogen source. Degradation was also assessed using gel electrophoresis and immunostaining for mucins showed the presence of degradation products. Furthermore, imaging fluorescently labeled PGM through confocal microscopy revealed significant intracellular fluorescence suggesting *C. albicans* can endocytose mucins. Growth, mucin degradation and invasion into the agarose-mucin matrix were reduced under anaerobic conditions.

**Conclusions:**

*C. albicans* was able to degrade and internalize gastric mucins under aerobic conditions, indicating a potential role for increased gut oxygen in CD to exacerbate this phenotype. Utilization of gut mucins by *C. albicans* may potentially disrupt the mucus barrier in CD, further exacerbating disease.

**Funding Agencies:**

CIHRWeston Family Foundation

